# Membrane Distribution and Activity of a Neuronal Voltage-Gated K+ Channel is Modified by Replacement of Complex Type N-Glycans with Hybrid Type

**DOI:** 10.4172/2168-958X.1000128

**Published:** 2017-10-31

**Authors:** M Kristen Hall, Douglas A Weidner, Sahil Dayal, Elena Pak, Alexander K Murashov, Ruth A Schwalbe

**Affiliations:** 1 Department of Biochemistry and Molecular Biology, Brody School of Medicine, East Carolina University, Greenville, USA; 2 Department of Microbiology and Immunology, Brody School of Medicine, East Carolina University, Greenville, USA; 3 Department of Physiology, Brody School of Medicine, East Carolina University, Greenville, USA

**Keywords:** Voltage-gated potassium channel, Transmembrane glycoprotein, Neuroblastoma, Cell surface glycan, Neurons, Outward ionic currents

## Abstract

Abnormal modifications in N-glycosylation processing are commonly associated with neurological disorders, although the impact of specific N-glycans on neuronal excitability is unknown. By replacement of complex types of N-glycans with hybrid types in neuroblastoma cells, we provide the first study that addresses how distinct N-glycan types impact neuronal excitability. Using CRISPR/Cas9 technology, NB_1, a clonal cell line derived from rat neuroblastoma cells (NB), was modified to create an N-glycosylation mutant cell line, NB_1 (-Mgat2), which expresses predominantly hybrid type N-glycans. Western and lectin blotting, flow cytometry, TIRF and DIC microscopy, and patch clamp studies were conducted. Lectin binding revealed the predominant type of N-glycans expressed in NB_1 (-Mgat2) is hybrid while those of NB and NB_1 are complex. Kv3.1 b-expressing cells with complex N-glycans localized more glycosylated Kv3.1b to the neurites than cells with hybrid N-glycans. Further the absence of N-glycan attachment to Kv3.1b was critical for sub-plasma distribution of Kv3.1b to neurites in primary adult mammalian neurons, along with NB cells. Replacement of complex type N-glycans with hybrid type hindered the opening and closing rates of outward ionic currents of Kv3.1 b-expressing NB cells. The lacks of N-glycan attachment hindered the rates even more but were not significantly different between the NB cell lines. Taken together, our evidence supports N-glycosylation impacts the sub-plasma membrane localization and activity of Kv3.1 b-containing channels. We propose that N-glycosylation processing of Kv3.1 b-containing channels contributes to neuronal excitability, and abnormal modifications in N-glycosylation processing of Kv3.1b could contribute to neurological diseases.

## Introduction

N-glycans are a part of glycoproteins and are classified as three major types: oligomannose, hybrid, and complex, each of which is composed of a common core sugar sequence [1]. N-acetylglucosaminyltransferases (GlcNAcTs, coded by Mgat genes) act to initiate the different branch points onto the common core, thereby creating the three major types of glycans [1]. N-glycosylation processing of proteins is vital for the folding, assembly, and trafficking of proteins, as well as cell recognition events [2,3]. Further the importance of N-glycosylation in multicellular organisms is evidenced in congenital disorders of glycosylation (CDG) of humans [4,5], and in mutant glycosylation mice [6–8]. As such, N-glycosylation is vital to the function of cells, and aberrant N-glycosylation processing can lead to numerous pathophysiological conditions.

Kv3 (Kcnc) voltage-gated potassium channels are essential to the effectiveness of high frequency firing neurons [9]. For instance, when one of the Kcnc genes was abolished in mice, neurons lacking Kv3 expression have broadened action potentials, and impaired high-frequency firing [10–12]. The Kv3 channels have two absolutely conserved occupied N-glycosylation sites [13,14]. The relevance of N-glycosylation site occupancy of both Kv3.1b and 3.1a proteins in cell function has been highlighted in studies that demonstrate N-glycans contribute to the opening and closing of Kv3 channels with Kv3.1b, as well as the spatial localization of Kv3.1b and 3.1a proteins in the plasma membrane, of Sf9 insect [15], CHO [16], and B35 neuroblastoma cells [17–19]. Further unoccupied sites of other Kv channels were shown to perturb channel activity [20–23] and membrane localization [22–25]. Thus, it is likely that unoccupied N-glycosylation sites of Kv channels would perturb neural excitability.

The primary aim of this paper was to examine the role of N-glycan processing, as well as N-glycan site occupancy, in altering the distribution of the Kv3.1b protein in the plasma membrane, along with the opening and closing rates of the Kv3 channels containing Kv3.1b. Here, we engineered a glycosylation mutant neuroblastoma cell line by substituting complex type N-glycans with hybrid type. Next, by expressing the glycosylated and unglycosylated forms of the Kv3.1b protein in the parental and glycosylation mutant cell lines, we observed differences in the outward ionic current of Kv3.1b-expressing cells. Also, we assessed the role of N-glycosylation site occupancy of Kv3.1b in sub-plasma membrane distribution in adult primary neurons. Taken together, results of this study elucidate the significance of N-glycan processing, and N-glycan site occupancy in controlling the firing properties of Kv3.1b-expressing neuronal-derived cells, thus potentially providing a better understanding of neurological diseases which affect mammalian brain.

## Materials and Methods

### Generation of NB clonal cell line with Mgat2 silenced

Individual rat B35 neuroblastoma cells (NB cells) were isolated in culture and grown to confluence to generate the NB_1 clonal cell line. CRISPR-Cas9 technology was used to silence the gene of interest [26]. The sgRNA oligonucleotides (5’-CACCGTTCCGCATCTACAAACGGA-3’ and 5’-AAACTCCGTTTGTAGATGCGGAAC-3’) were used to create the neuroblastoma cell line with Mgat2 silenced thus predominantly expressing hybrid N-glycans [27]. In brief, the sgRNA oligonucleotides were selected using the Zi-Fit Targeter software [28,29]. After phosphorylation and annealing of oligonucleotide, double-stranded gRNA molecules were cloned into the pSpCas9(BB)-2A-Puro vector (Addgene plasmid ID: 48139) and sequencing confirmed. The expression vector was transfected into NB_1 cells using lipofectamine 2000 [30]. Transfected cells were selected with 0.25 μg/mL puromycin for 48 h. Genomic DNA was isolated from numerous cell clones and Mgat2 silencing was confirmed by DNA sequencing of the targeted genomic region. The cell line is referred to as NB_1 (-Mgat2).

### Cell culture and stably transfected cell lines of neuroblastoma

NB cells were obtained from American Type Culture Collection (Manassas, VA, USA) and grown in DMEM containing 10% FBS at 37°C in a 5% CO_2_ atmosphere. Kv3.1b was ectopically expressed in the NB_1 and NB_1 (-Mgat2) cell lines, as previously described for NB [18]. In brief, cells of 70-80% confluency were transfected with neomycin selectable expression plasmids encoding the α-subunit of Kv3.1b. Cells were maintained at 37°C under 5% CO_2_ in DMEM (GE, Logan, UT, USA) supplemented with 10% fetal bovine serum, 50 U/mL penicillin and 50 μg/mL streptomycin (Gemini BioProducts, West Sacramento, CA, USA).

### Primary neuronal cell isolation and culture

Experiments were performed on adult C57 black mice (4-8 weeks of age) according to the guidelines of the animal care and use committee of East Carolina University, an AAALAC-accredited facility. Mixed cortical/hippocampal neurons were isolated and cultured with minor modifications [31]. In brief, cells were released from dissected hippocampi and cortex by incubation with papain (Worthington Biochemical Corporation, Lakewood, NJ, USA) by gentle trituration. Subsequently, neuronal fractions from the cells were separated utilizing OptiPrep density gradient (Sigma, St. Louis, Mo, USA) and seeded in Neurobasal medium containing B27 supplement, 0.5 mM glutaMax, gentamycin, mouse FGF2, and PDGFbb (10 ng/mL) growth factors (Thermofisher Scientific, Waltham, MA, USA) onto 35 mm poly-D-lysine coated glass bottom dishes (MatTek, Ashland, MA,USA) and incubated at 37°C. Upon neurite and synaptic formation (7-10 DIV), cells were transfected with glycosylated (wt) and unglycosylated (N220/9Q) forms of Kv3.1b using Lipofectamine^®^ 2000 reagent (Thermofisher Scientific, Waltham, MA, USA) as described [18]. Cells greater than 48 h post transfections were utilized for live cell total internal reflection fluorescence (TIRF) microscopy studies, and to make whole cell lysates for western blots.

### Lectin binding analysis by flow cytometry

Cells were incubated with 10 μg/mL of a single fluorescein tagged lectin (Vector Laboratories, Inc., Burlingame, CA, USA) *Phaseolus vulgaris* leucoagglutinin (L-PHA), *Galanthus nivalis* lectin (GNL), *Phaseolus vulgaris* erythroagglutinin (E-PHA), or concanavalin A (ConA) for 15 min at room temperature. A FACS Vantage flow cytometer (Becton Dickinson Biosciences, San Jose, CA) was used with 488 nm laser excitation and emission centred at 530 nm to acquire fluorescence intensity. Mean fluorescence values were determined from histogram plots of fluorescence emission.

### Glycosidase digestions

Total membranes were isolated from cells as previously described [18]. Cells were homogenized in lysis buffer (10 mM Tris, pH 7.4; 250 mM sucrose, 5 mM EDTA; protease inhibitor cocktail set III (Calbiochem, San Diego, CA, USA) 1:500). After centrifugation of lysate, the supernatant was collected and subsequently centrifuged at 100,000× g for 1 h. Pellet was resuspended in lysis buffer and protein concentration was determined by Lowry assay. Glycosidase digestions of total membranes (5 g/L) were treated with 20 U/μL PNGase F, 50 U/μL Endo H and 0.83 U/μL neuraminidase in supplied buffers (New England Biolabs, Ipswich, MA, USA). Reactions were incubated overnight at 37°C and followed by the addition of reducing SDS-PAGE sample buffer.

### Western and lectin blots

Kv3.1b total membrane samples for western blotting and whole cell lysates for lectin blotting were separated by 10% SDS-PAGE gels for 1.7 h at 20 mA. Electrophoresed proteins were transferred to PVDF membranes (Millipore, Billercia, MA, USA) for 2.5 h at 250 mA. Blots were incubated and developed as described previously [18,27]. Mouse anti-Kv3.1 antibody (Neuromab, Davis, CA, USA) was utilized to detect Kv3.1b. Lectin blots were probed with Biotin-conjugated L-PHA, E-PHA, or GNL (Vector Laboratories, Burlingame, CA, USA).

### TIRF microscopy

Kv3.1b transfected cells were seeded onto 35 mm poly-L-lysine coated glass bottom dishes (MatTek, Ashland, MA, USA) and incubated for 18-20 h. TIRF, differential interference contrast (DIC) and wide-field images of the cells were captured. Live cells were excited with an argon laser beam of wavelength 488 nm. An Apo 60× 1.45 objective attached to an Olympus IX-71 microscope (Olympus, Center Valley, PA, USA) was utilized and images were captured with an ORCA R2 deep cooled mono CCD camera. Detection settings were kept constant. Exposure time of 1000 ms was used for data analysis. The shutters, filters, camera and data acquisition were controlled by Cell^^^TIRF Control 1.1 and Metamorph for Olympus Basic software. Particle number, area of particle, and mean intensity of particles in total cell, outgrowth, and cell body was measured using Image J software.

### Whole cell recordings

The whole cell configuration of the patch clamp technique was used to obtain electrophysiological measurements from Kv3.1b transfected NB_1 and NB_1 (-Mgat2) cells as previously described [15,17,32]. In brief, an external bath solution composed of (in mM): 5 potassium aspartate, 135 sodium aspartate, 1 MgCl_2_ hexahydrate, 10 Mes, 60 mannitol (pH 7.1) and 300-312 mOsm. Intracellular solution contained (in mM0: 140 potassium aspartate, 10 EGTA, 5 MgCl_2_ hexahydrate, 10 HEPES, 50 mannitol (pH 7.2) and 320-340 mOsm was utilized. Compensation of cell capacitance and series resistance was ensured during the experiments. Currents were sampled at 10 kHz and then filtered at 1 kHz. Whole cell recordings were analyzed when membrane seal resistance was about 1 GΩ, and maximum current amplitude was > 880 pA. The endogenous currents were small relative to the various Kv3.1b transfected NB cell lines, similar to those reported for the NB (aka B35) cell line [17]. The current densities in non-transfected NB_1 and NB_1 (-Mgat2) were 2.7 ± 1.0 (n=4) and 1.5 ± 1.2 (n=6), respectively, while those for Kv3.1b transfected NB_1 and NB_1 (-Mgat2) were 245 ± 37 (n=20) and 258 ± 33 (n=18), respectively. Whole cell recordings were obtained from activation and deactivation voltage clamp protocols as described in results.

### Data analysis

Digitized whole cell current recordings from activation protocols were used to determine the potential at which the conductance was half maximal (V_0.5_), and the slope factor (dV) from normalized conductance-voltage plots, and also, to acquire the time needed for the current to rise from 10% to 90% of its peak current at a given test potential (rise time). Conductance-voltage relationships were fitted with a Boltzmann equation of the form: G=G_max_/[1+exp(V_0.5_−V_m_)/dV] where dV represents the slope factor, V_m_ stands for the test potential, V_0.5_ is the potential at which the conductance was half maximal, G is the conductance and G_max_ is the maximal conductance. Whole cell currents at various test potentials from deactivation voltage protocols were fitted with a single exponential to report mean deactivation time constant (tau_off_). Origin 7.5 (OriginLab Corporation, Northampton, MA, USA) was used for data analysis and graphics. Data are presented as the mean ± S.E. or as indicated. Student’s t-test was utilized to evaluate the statistical comparison of two groups while one-way ANOVA with Bonferroni adjustments was used for more than two groups as indicated.

## Results

### Engineering of the glycosylation mutant cell line

**Figure 1:**
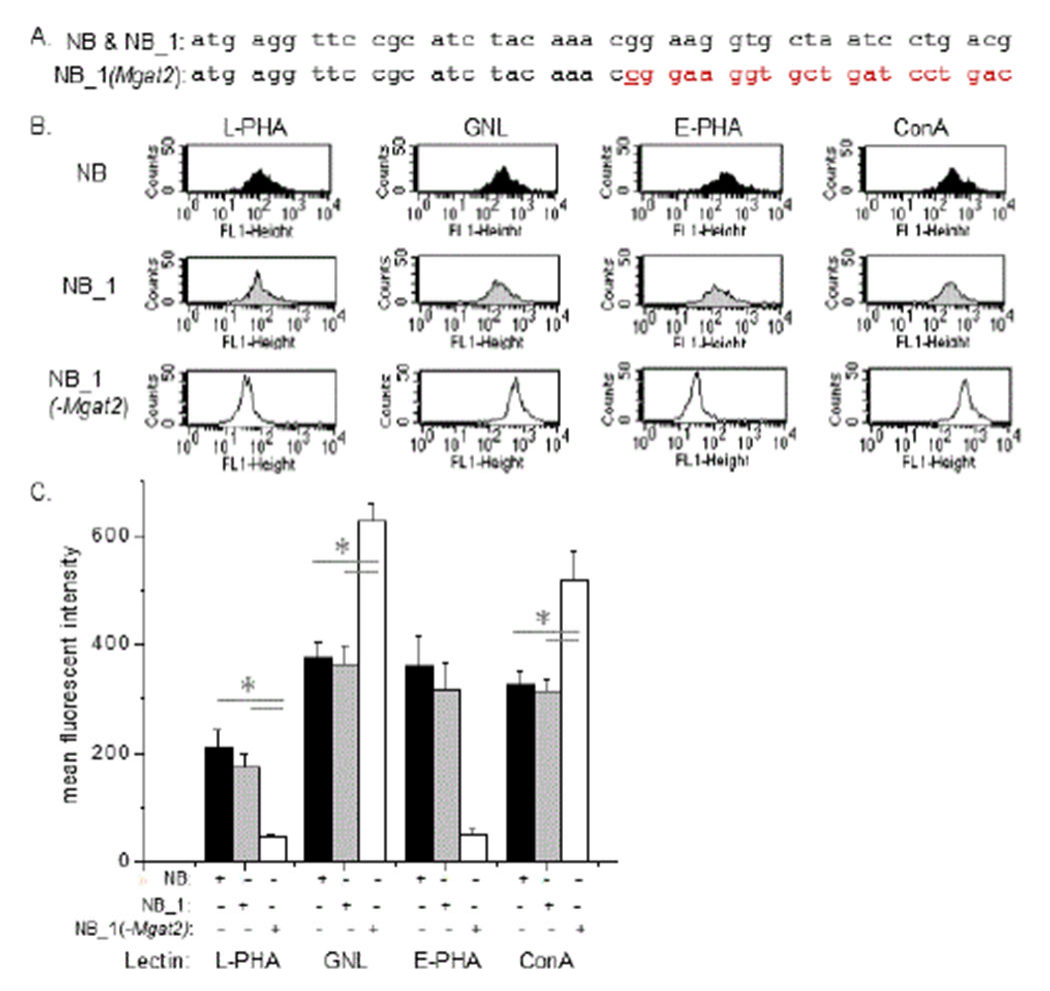
Characterization of neuroblastoma cell lines with differences in the N-glycosylation pathway. CRISPR/Cas9 technology was employed to silence the Mgat2 gene in a clonal cell line (NB_1) isolated from NB cells. The coding sequence (CDS) of the Mgat2 gene from 1 to 42 is shown for the NB cell line which matched the NB_1 cell line, and the isolated clonal cell line, NB_1 (-Mgat2) (A). NB_1 (-Mgat2) has the Mgat2 gene silenced, resulting from insertion of c after the 22nd nucleotide. This inserted nucleotide is denoted in bold red font and the red font reveals the different codons in the sequence. Representative flow cytometry plots of fluorescently labelled lectins bound to NB (top panels), NB_1 (middle panels), and NB_1 (-Mgat2) (bottom panels) cell lines (B). The lectins were L-PHA (left panels), GNL (left middle panels), E-PHA (right middle panels), and ConA (right panel) (B). Mean fluorescence values of the various neuroblastoma cell lines (C) were ascertained from 6 separate lectin binding experiments for L-PHA, GNL, and E-PHA, and 4 experiments for Con A. (*) denote that NB_1 (-Mgat2) was significantly different from NB and NB_1 at a probability of P<0.05 using One-way ANOVA with Bonferroni adjustments.

The role of N-glycosylation processing of Kv3.1 in modifying neuronal excitability was examined by replacement of complex N-glycans with hybrid type N-glycans in rat neuroblastoma (NB) cells. To reduce heterogeneity, a clonal NB cell line, (NB_1), was established from parental NB cells, and subsequently utilized as the clonal cell line for creating a cell line with the Mgat2 gene silenced (NB_1 (-Mgat2). The N-glycosylation mutant cell line was engineered using the CRISPR/Cas9 method (26). The NB_1 (-Mgat2) cell line was identified and confirmed by DNA sequencing of nine separate cell clones. The coding sequence of the Mgat2 gene had a C residue inserted after the 22^nd^ nucleotide residue which results in a frame shift mutation (Figure 1A). The mutation presents nonsense amino acid sequence and numerous early premature stop codons. An alternative start site occurs at amino acid residues 98 of the wild type coding sequence of GlcNacT-II.

### Predominant type of N-glycans in neuroblastoma cell lines

N-glycans expressed on the cell surface of NB, NB_1, and NB_1 (-Mgat2) cell lines were characterized using various fluorescent lectins with a flow cytometric assay. *Phaseolus vulgaris* Leucoagglutinin (L-PHA) has higher affinity for tri- and tetra-antennary complex type N-glycans, *Phaseolus vulgaris* Erythoagglutinin (E-PHA) has a higher affinity for complex type N-glycans with bisecting N-acetylglucosamine, concanavalin A (ConA) binds tightly to oligomannose and hybrid type N-glycans and *Galanthus nivalis* Lectin (GNL) has a higher affinity for α-mannose residues [33,34]. Representative flow cytometry histograms showed the NB, NB_1, and NB_1 (-Mgat2) cells lines with the different lectins, as indicated (Figure 1B). The flow cytometry plots showed that L-PHA and E-PHA lectins bind to the cell surface of NB_1 (-Mgat2) cells with much less affinity than both NB and NB_1. On the other hand, both ConA and GNL bound at a much higher level to NB_1 (-Mgat2) cells than either NB or NB_1 cells. Analysis from at least 4 separate experiments clearly illustrated that NB and NB_1 cells had remarkably similar cell binding interactions for each of the 4 lectins bound to their cell surface while NB_1 (-Mgat2) displayed much different binding affinities for each of the tested lectins (Figure 1C).

**Figure 2:**
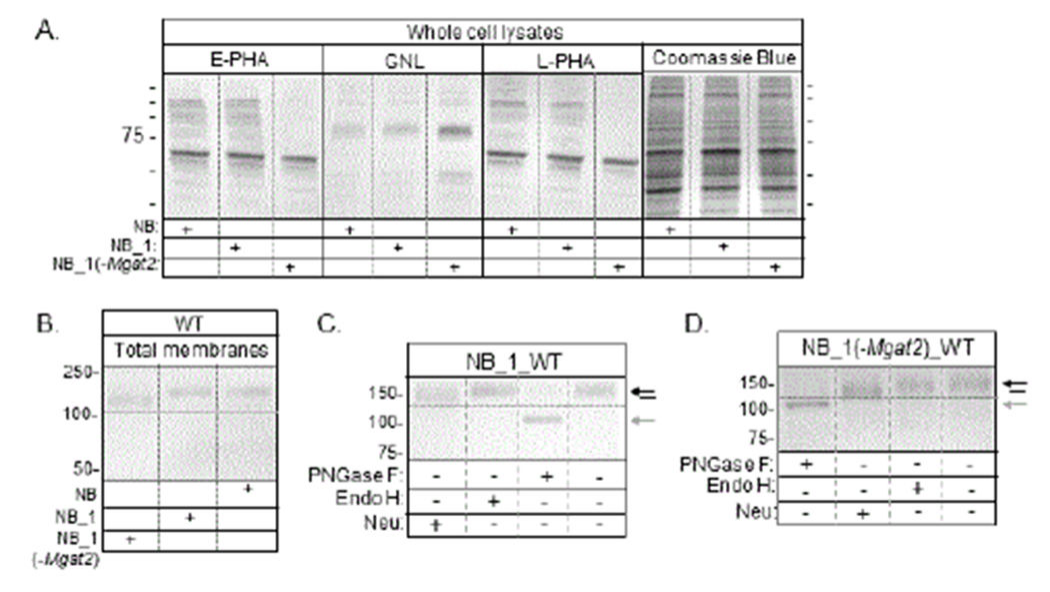
Establishment of the predominant type of glycans at the cell surface and correlation of changes in electrophoretic migration of Kv3.1b in neuroblastoma cell lines. Lectin blots of separated proteins from whole cell lysates of NB, NB_1 and NB_1 (-Mgat2) cell lines probed with E-PHA, GNL and L-PHA, and an SDS gel of proteins stained with coomassie blue (A). Lectin blots were reproduced at least 3 times. Plus signs indicate the sample loaded in each well. Lines next to blot and gel represent one of the six molecular weight standards in kDa: 250; 150; 100; 75; 50; and 37 from top to bottom. A western blot of ectopically expressed Kv3.1b in total membranes from transfected NB, NB_1 and NB_1 (-Mgat2), as denoted by the plus sign (B). Total membranes of Kv3.1b transfected NB_1 (C) and NB_1 (-Mgat2) (D) cell lines digested (+) and undigested (−) with neuraminidase (neu), Endo H and PNGase F. Black and grey arrows point to glycosylated and unglycosylated Kv3.1 proteins, respectively. Lines point to glycosylated Kv3.1 protein with siailic acid residues removed. Horizontal dashed lines adjacent to immunobands on blot were used to assist in visualization of electrophoretic shifts. The numbers adjacent to the Western blots represent the Kaleidoscope markers (in kDa).

The repertoire of N-glycans expressed by the cell lines were further characterized by lectin blotting. E-PHA and L-PHA lectins had highest affinity for glycans of proteins produced in NB and NB_1 cells while GNL had the highest affinity for those produced in NB_1 (-Mgat2) cells (Figure 2A).

The Coomassie blue stained gel confirmed the levels of protein loaded per well for the lectin blots were quite similar. In all cases, the binding affinities of the lectins for glycans attached to proteins expressed in NB and NB_1 cell lines were virtually identical while NB_1 (-Mgat2) had a novel lectin binding profile. Overall, the lectin binding studies revealed that NB and NB_1 cells predominantly express complex type N-glycans with significant levels of bisecting type N-glycans while complex type of N-glycans appeared to be considerably reduced, if not absent, in the NB_1 (-Mgat2) cell line. The difference in binding of GNL, as well as ConA, between NB_1 (-Mgat2) cells and either NB or NB_1 cells was quite similar to the lectin binding ratio of Pro-5 (expresses predominantly complex N-glycans) to K16 (expresses predominantly hybrid N-glycans) CHO cell lines as found in our previous study [27]. Further, it was unlike the lectin binding ratio of Pro-5 to Lec1 (expresses oligomannose N-glycans) CHO cell lines [27]. Taken together, our results support that the N-glycan predominantly expressed by NB_1 (-Mgat2) was hybrid N-glycans, and furthermore that the Mgat2 gene was silenced in the NB_1 (-Mgat2) cell line.

### Expression of Kv3.1b with hybrid type N-glycans

Earlier studies show that the N-glycosylated α-subunit protein of Kv3.1b/a channels has a different distribution than their unglycosylated counterparts in B35 neuroblastoma [18] and CHO [16] cell lines. In the current study, we evaluated the distribution of ectopic expression of GFP tagged Kv3.1b in stably transfected NB cell lines that express different types of N-glycans. A western blot of Kv3.1b in total membranes from the various cell lines showed that Kv3.1b protein expressed in NB_1 (-Mgat2) cells had a faster electrophoretic migratory rate than that from NB_1 and NB (Figure 2B). To verify that the N-glycans attached to Kv3.1b expressed in NB_1 cells are of complex type N-glycans, as previously shown in NB cells [17], membranes from NB_1 were treated with various glycosidases (Figure 2C). As expected, immunoband shifts were detected with PNGase F (cleaves all types of N-glycans) and neuraminidase (Neu, removes sialic acid residues at the end of chains) treatment but not endoglycosidase H (Endo H, cleaves oligomannose type N-glycans) treatment. Kv3.1b protein in total membranes from NB_1 (-Mgat2) cells also underwent immunoband shifts when treated with PNGase F and a small shift upon treatment with neuraminidase while an immunoband shift was absent when treated with endoglycosidase H (Figure 2D). These studies revealed that N-glycans attached to Kv3.1b in the NB_1 cell line were complex type N-glycans, similar to the NB cell line, while hybrid type N-glycans were associated with Kv3.1b in the NB_1 (-Mgat2) cell line. These findings, along with the lectin binding studies, verified that the NB_1 and NB_1 (-Mgat2) cell lines process N-glycans to complex and hybrid types, respectively.

### Types of N-glycans influence sub-plasma membrane localization of Kv3.1b

**Figure 3:**
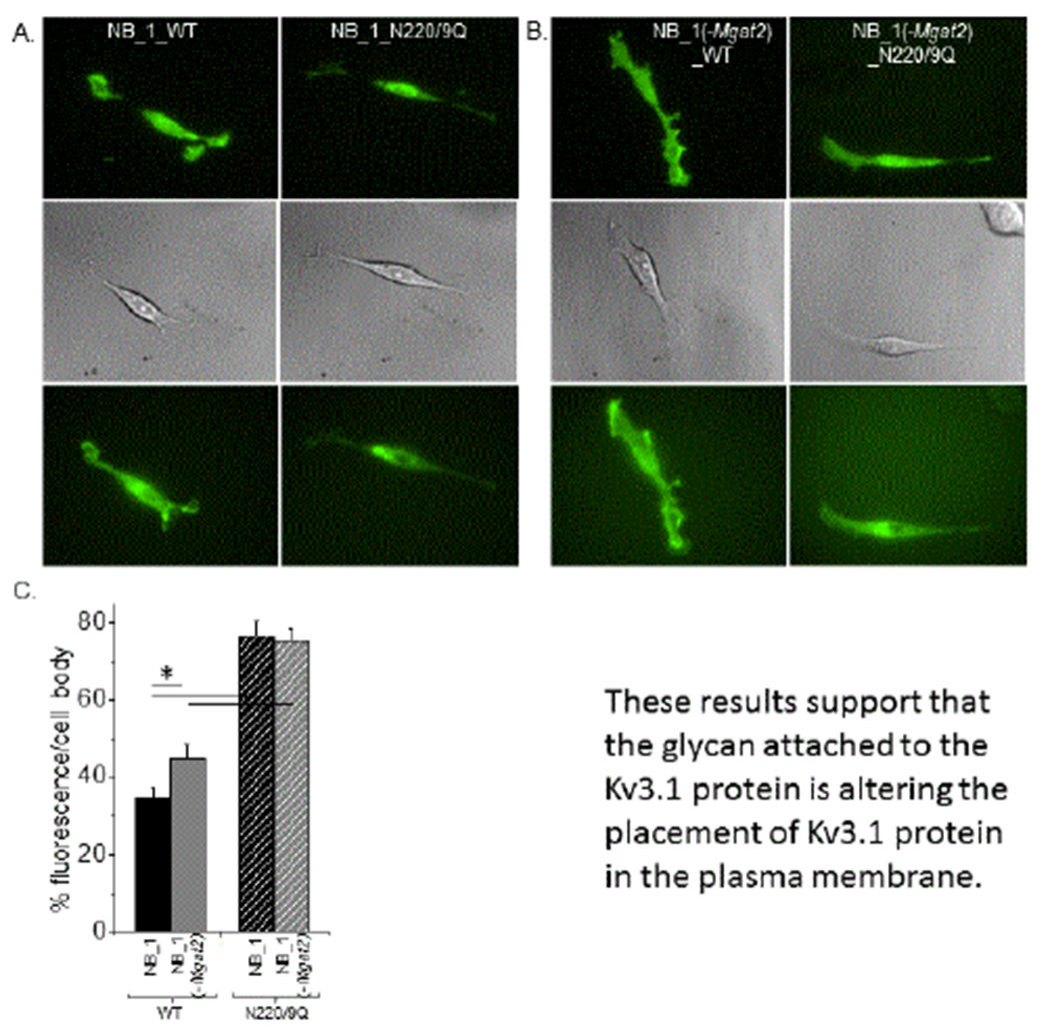
Subdomain localization of Kv3.1b in neuroblastoma cells with different N-glycosylation pathways. Microscopy images were obtained in TIRF (upper panels), DIC (middle panels) and wide-field (lower panels) modes for various forms of EGFP tagged Kv3.1b protein. Glycosylated (left panels) and unglycosylated (N220/9Q; right panels) Kv3.1b expressed in NB_1 (A) and NB_1 (-Mgat2) (B). Representative scale bar (5 μM) was similar for all acquired images. Percent fluorescence in the cell body of NB_1 cells expressing either glycosylated or unglycosylated Kv3.1b relative to NB_1 (-Mgat2) cells expressing the two different forms of Kv3.1b (C). Experiments were performed on at least 3 different days and at least 45 cells were examined in each group. Asterisks indicate significant differences in mean values at a probability of P<0.05 using unpaired student T-test.

Micrographs were acquired from live NB_1 (Figure 3A) and NB_1 (-Mgat2) (Figure 3B) cells expressing glycosylated (WT) or unglycosylated (N220/9Q) Kv3.1b protein tagged with EGFP to examine distribution of the Kv3.1b protein as a function of N-glycan structure. High contrast images of NB_1 and NB_1 (-Mgat2) cells expressing various forms of Kv3.1b proteins at the plasma membrane were acquired using total internal reflection fluorescence (TIRF) microscopy (top panels). The fluorescent signal was detected in the cell body and outgrowths for the glycosylated Kv3.1b protein while the signal appeared to accumulate in the cell body for the aglycoforms. The localization of the fluorescent signal to a given subdomain was verified using the accompanied differential interference contrast (DIC) images, which were acquired in virtually identical planes (middle panels). The wide-field images, acquired at a similar depth as the TIRF images, revealed the placement of the nucleus in the cells, and they have a much more diffuse signal than the TIRF images (bottom panels). Comparison of the images from the various microscopy modes supported that the fluorescent signal in the TIRF mode was from EGFP tagged Kv3.1b in or near the plasma membrane. The percent of fluorescence in cell body was lowest for glycosylated Kv3.1b in NB_1 cells, and in both cases, more Kv3.1b protein was localized to the cell body for the unglycosylated forms (Figure 3C). These results indicated that substitution of complex type N-glycans with hybrid type in NB cells causes more Kv3.1b protein to accumulate in the cell body. Further, these results verified our earlier study [18] that the absence of N-glycosylation of the Kv3.1b protein results in accumulation of the Kv3.1b protein to the cell body.

**Figure 4:**
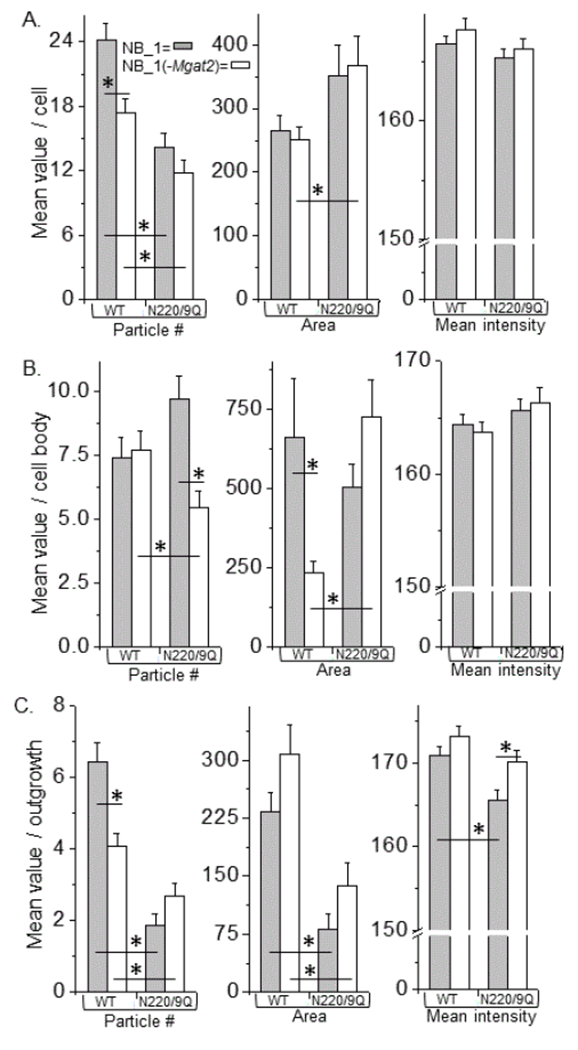
Comparison of neuronal membrane distribution of Kv3.1b with predominant expression of hybrid or complex types of N-glycans. Fluorescent particles from EGFP tagged glycosylated (WT) and unglycosylated (DM) Kv3.1b proteins in NB_1 and NB_1 (-Mgat2) cells were analyzed in or near plasma membrane of cell (A), cell body (B), and outgrowth (C). Number (left panels), area (middle panels) and mean intensity (right panels) of particles were compared between the glycosylated Kv3.1b proteins, as well as the unglycosylated proteins, in each cell line. The glycosylated and unglycosylated Kv3.1b proteins expressed in the same cell line were also compared. Experiments were performed on at least 3 different days and at least 31 cells were examined in each group. Asterisks indicate significant differences in mean values at a probability of P<0.05 using unpaired student T-test.

To identify changes in the distribution of the various forms of the glycosylated Kv3.1b protein (WT) and their unglycosylated counterparts (N220/9Q), fluorescence particle analysis was conducted via Image J software (Figure 4). Mean values of number (left panels), area (middle panels), and mean intensity (right panels) of particles in adhered membrane from a cell (A), cell body (B) and outgrowth (C) are shown. The area and fluorescence intensity signal of the particles in the adhered membrane for glycosylated Kv3.1b produced in NB_1 and NB_1 (-Mgat2) were quite similar; however, the number of particles was significantly greater in NB_1.

All three parameters of the particles for the aglycoform expressed in NB_1 and NB_1 (-Mgat2) were similar. In both cases, more particles of smaller size were detected for the glycosylated form of Kv3.1b relative to the unglycosylated form in both the NB_1 and NB_1 (-Mgat2) cell lines. Overall, these results supported that the level of Kv3.1b protein expressed in the cell lines were quite similar. Next, the various parameters obtained from particle analysis of glycosylated and unglycosylated Kv3.1b in the cell body and outgrowths were compared. In terms of the cell body, the glycosylated form of Kv3.1b produced much larger particles in NB_1 than those in NB_1 (-Mgat2), while the other two parameters were quite similar. Glycosylated Kv3.1b in NB_1 had more particles in an outgrowth than that produced in NB_1 (-Mgat2), while the other parameters were comparable. When comparing the unglycosylated Kv3.1b protein, more particles were detected in cell body and less dense particles were found in the outgrowth of the unglycosylated Kv3.1b expressed in NB_1 compared to that in NB_1 (-Mgat2). Thus, these results indicated that the type of N-glycan attached to Kv3.1b and the type of N-glycan at the cell surface can impact the spatial arrangement of Kv3.1b in the plasma membrane of neuronal derived cells.

### Localization of Kv3.1b in primary neurons is influenced by N-glycosylation site occupancy

**Figure 5:**
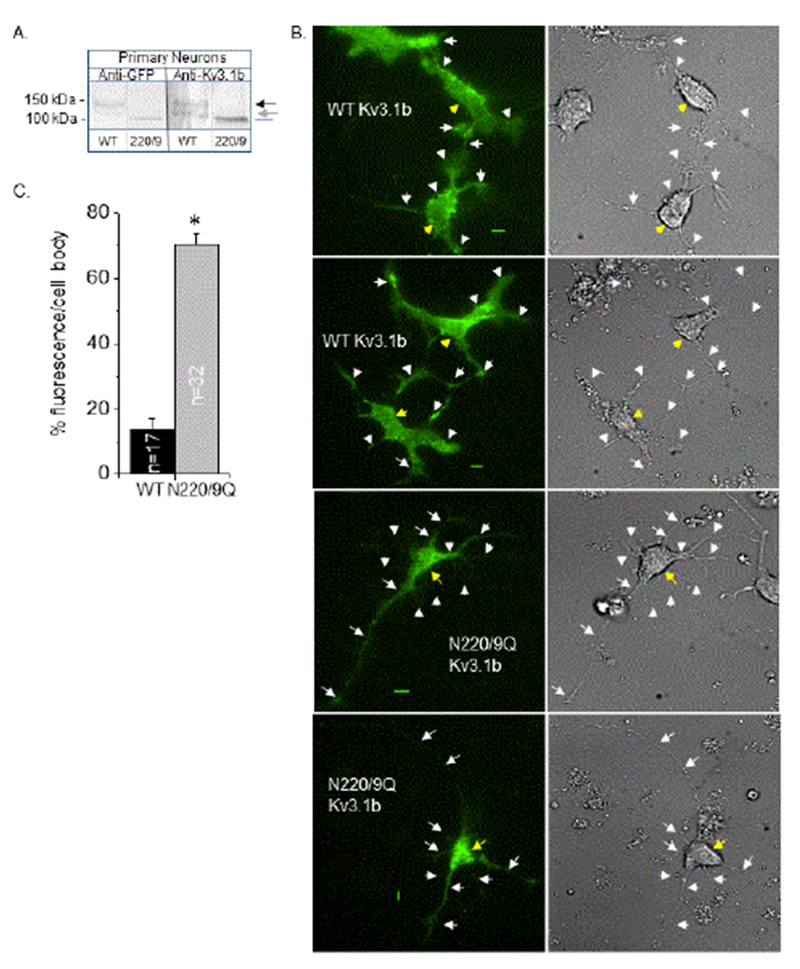
Characterization of ectopic expression of glycosylated and unglycosylated forms of the Kv3.1b protein in adult primary neurons. Western blots of EGFP tagged Kv3.1b in whole cell lysates from adult primary neurons transfected with glycosylated (WT) and unglycosylated (220/9) forms of the Kv3.1b protein (A). Arrows denote glycosylated forms of the Kv3.1b protein, and gray line represents unglycosylated Kv3.1b protein. Representative TIRF (left panels) and DIC (right panels) images acquired in similar planes are shown for neurons expressing glycosylated (top two panels) and unglycosylated (bottom two panels) forms of the Kv3.1b protein (B). White and yellow arrows point to outgrowths and cell body, respectively. Representative scale bar (5 μm) was identical for image pairs. Percent of fluorescence intensity in cell body was determined to quantify differences between the level of glycosylated and unglycosylated Kv3.1b in cell body (C). Asterisk denotes significant differences in mean values at a probability of P<0.000001 using student t-test. Experiments were performed on at least 3 different days and at least 32 cells were examined in each group.

Glycosylated (WT) and unglycosylated (220/9) Kv3.1b proteins tagged with EGFP were transiently expressed in rat adult primary neurons. The tagged proteins were detected with anti-GFP and anti-Kv3.1b antibodies by the Western blotting technique (Figure 5A). As expected, a doublet was detected for the WT protein, and a faster migrating immunoband for the 220/9 protein. Based on our previous studies [17,30], the upper and lower immunobands of the doublet are glycosylated Kv3.1b protein with complex and oligomannose types of N-glycans, respectively and the immunoband for the 220/9 protein represents the aglycoform. TIRF (left panels) and DIC (right panels) images were acquired for the glycosylated (upper two panels) and unglycosylated (lower panels) Kv3.1b protein (Figure 5B). These images demonstrated that the glycosylated and unglycosylated forms of the Kv3.1b protein were distributed to both the outgrowths and cell body of adult primary neurons, but much more Kv3.1b protein was observed in the outgrowths for the glycosylated form. This difference was substantiated by measuring the amount of fluorescence in cell body relative to that in a neuron (Figure 5C). Taken together, these results demonstrated that N-glycosylation processing of the Kv3.1b protein enhances its delivery to the outgrowths of adult neurons.

### Kinetics of outward ionic currents

**Figure 6:**
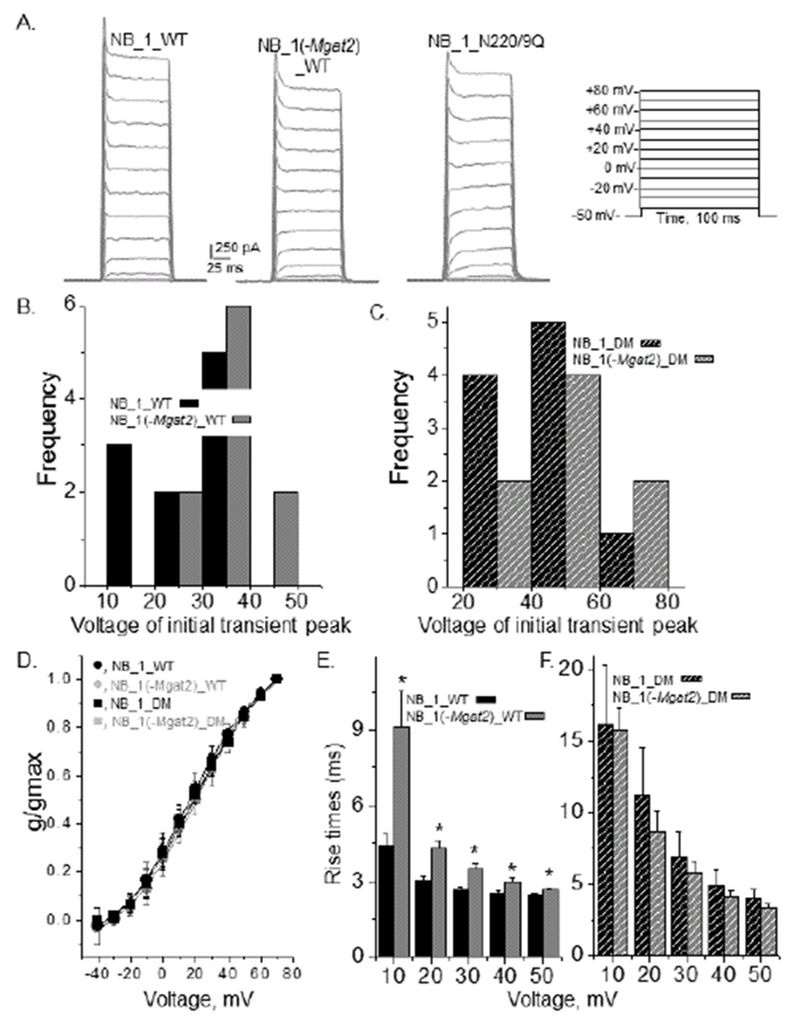
Activation kinetics of different glycosylated and unglycosylated forms of Kv3.1b in neuroblastoma cells. Whole cell currents were elicited from the indicated voltage protocol for glycosylated Kv3.1b expressing NB_1, and NB_1 (-Mgat2) cell lines, and the unglycosylated Kv3.1b expressing NB_1 cell line (A). All-points-histograms show the lowest voltage at which the transient peak was observed in each whole cell recording for glycosylated (B) and unglycosylated (C) Kv3.1 in the two cell lines. Conductance-voltage (g/g_max_) curves for glycosylated and unglycosylated Kv3.1 channels in NB_1 and NB_1 (-Mgat2) (D). Data are presented as the mean ± S.D. Rise times of glycosylated (E) and unglycosylated (F) Kv3.1 proteins expressed in the given cell lines. Experiments were performed on at least 3 different days and at least 8 cells were examined in each group. Asterisk denotes significant differences in mean values at a probability of P<0.05 using student t-test.

Whole cell recordings were obtained from NB_1 and NB_1 (-Mgat2) cells (Figure 6A) stably expressing glycosylated (Kv3.1b) or unglycosylated (N220/9Q) Kv3.1b, as indicated. The predominant type of current expressed was of non-inactivating with transient peaks. However, the transient peaks were observed at different test potentials. The first observation of a transient peak from currents elicited by the activation voltage protocol was detected at a lower test potential for NB_1_WT than that for NB_1 (-Mgat2)_WT (Figure 6B) while comparison of those for NB_1_DM than NB_1 (-Mgat2)_DM cell lines were quite similar (Figure 6C). In both cases, the initial transient peaks were detected at lower test potentials for glycosylated Kv3.1b than their unglycosylated counterparts. Boltzmann plots of the activation currents revealed that the voltage-dependence of the channel activation were quite similar (Figure 6D).

**Figure 7:**
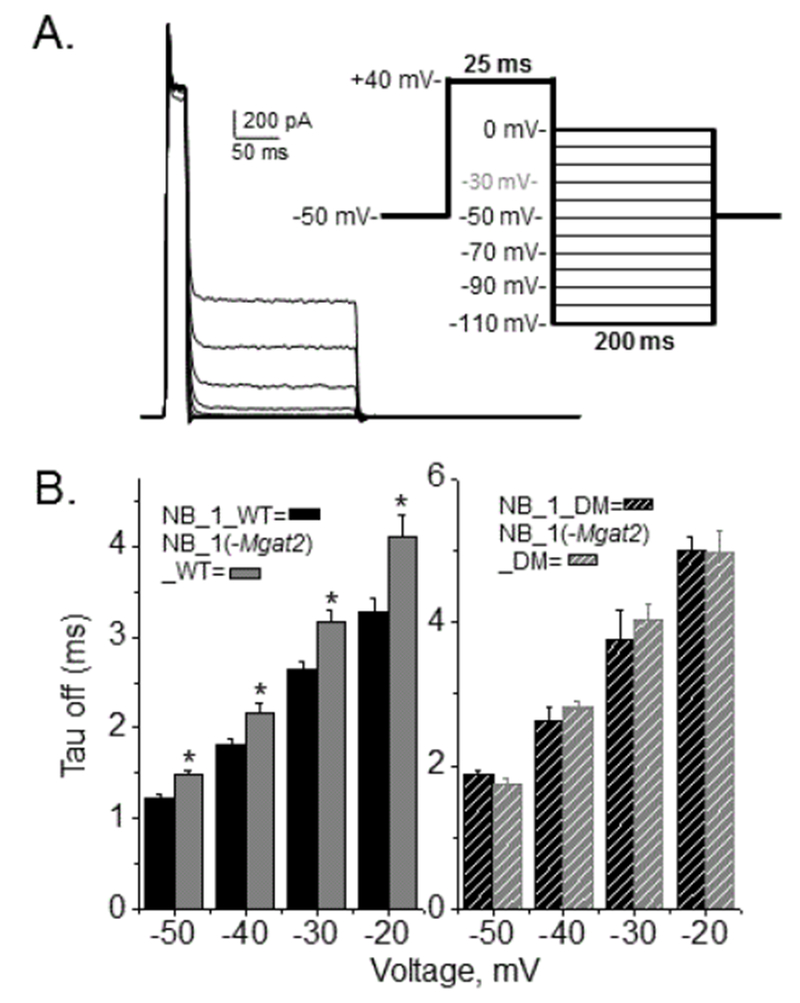
Deactivation rates of ionic currents for the Kv3.1b channel expressed in NB cell lines with different N-glycosylation pathways. Deactivation currents were elicited from the accompanied voltage protocol for NB_1 cells expressing glycosylated Kv3.1b (A). Black line denotes current at −30mV. Bar graphs revealing deactivation rates of glycosylated KV3.1 (B, left panel) and unglycosylated Kv3.1 (B, right panel) among the cell lines. Experiments were performed on at least 3 different days and at least 5 cells were examined in each group. Asterisk denotes significant differences in mean values at a probability of P<0.05 using student t-test.

However, expression of glycosylated Kv3.1 in NB_1 produced faster rise times than NB_1 (-Mgat2) expressing glycosylated Kv3.1 (Figure 6E). On the other hand, the unglycosylated Kv3.1b channels expressed in the NB cell lines had similar rise times (Figure 6F). Further, their rise times were much slower than their glycosylated counterparts. The currents stimulated by the deactivation voltage protocol of the various NB cell lines expressing Kv3.1b appeared similar (Figure 7A). However, examination of the deactivation rate constants (tau off) were faster for the glycosylated Kv3.1b channel expressed in the NB_1 cell line than that in the NB_1 (-Mgat2) cell line (Figure 7B). Expression of the unglycosylated Kv3.1b protein in either the NB_1 or NB_1 (-Mgat2)_DM cell lines were remarkably similar and in both cases, they were much slower than the cell lines expressing their glycosylated counterparts (Figure 7B). Therefore, these results revealed that complex and hybrid types of N-glycans altered the kinetics of the outward currents of the Kv3.1b channels, and furthermore, indicated that prevention of N-glycosylation processing decreased the opening and closing rates of the Kv3.1b channel. The present results also verified our prior studies [15,17] that N-glycan site vacancy of the Kv3.1 channel impedes activation and deactivation rates of the channel.

## Discussion

In this study, we found that substitution of complex type N-glycans with hybrid type in the NB cell line altered the placement of the Kv3.1b protein in the plasma membrane, and the opening and closing rates of Kv3 channels containing Kv3.1b. This was investigated by silencing the Mgat2 gene in rat B35 neuroblastoma (NB) cells by employment of the CRISPR/Cas9 technology. Previously, it was shown that this NB cell line expresses Kv3.1b protein with complex type N-glycans [17,30] as observed in adult mammalian brain [14,30]. To create the novel NB cell line that expresses predominantly hybrid type N-glycans, we utilized a gRNA pair conserved across species that we previously employed to silence the Mgat2 gene in the CHO Pro-5 cell line [27]. Cell surface glycan repertoires obtained from lectin binding studies, along with lectin binding profiles from parental and glycosylation mutant CHO cell lines [27,34,35], supported that the predominant type of N-glycans expressed in NB_1 (-Mgat2) was hybrid while those of parental (NB) and clonal (NB_1) NB cell lines were complex type. Therefore, the conserved gRNA pair used for the CRISPR/Cas9 method in creating cell lines with Mgat2 silenced was quite efficient, and subsequently can provide mutant N-glycosylation cell lines to identify the roles of hybrid type N-glycans in cell function.

Neuronal populations typically express multiple types of Kv channels with distinct sub-plasma membrane distributions which greatly impact their firing properties [36]. An identified contributor of modifying sub-plasma membrane pools of Kv3.1 channels is occupancy of the N-glycosylation sites of Kv3.1b and Kv3.1a [18,19]. Here, we showed that the substitution of complex type N-glycans with hybrid type modified the number and size of Kv3.1b clusters in the membranes of the cell body and neurites of NB cells, indicating that processing of the N-glycans also contributed to distinct sub-plasma membrane distributions of the Kv3 channels. Secondly, we demonstrated that vacancy of the N-glycosylation sites of Kv3.1b caused accumulation of Kv3.1b to the cell body in primary adult mammalian neurons. The distribution of Kv3.1b between the cell body and neurites of NB cells which processed N-glycans to complex type was more similar to that of primary neurons as less Kv3.1b was in the cell body. Since primary neurons mimic the *in vivo* distribution of Kv channels in neuronal membranes [37–41], our findings strongly supported that N-glycosylation processing provides a mechanism for regulating the sub-plasma membrane distribution of Kv3 channels in the mammalian brain.

Controlling the duration of a nerve impulse, as well as repetitive nerve firing, is critical for normal bodily functions. Kv3 channels can shorten the nerve impulses to a further degree than other Kv channels, and at least one of the four Kv3 genes are expressed in fast-spiking neurons of brain [9]. Earlier studies show that Kv3 channels containing Kv3.1b α-subunits with both N-glycosylation sites occupied have faster opening and closing rates than when sites are vacant [17]. It is also demonstrated that changing the N-acyl side chain of sialic acid of the N-glycans attached to Kv3.1b modify the activation rate of glycosylated Kv3.1b channels [32]. In the current study, we showed that the activation and deactivation rates of the outward ionic currents of Kv3.1b-expressing NB cells were slowed when complex type N-glycans were replaced with hybrid type N-glycans. Since the N-glycan pools in mammalian brain contain prevalent levels of complex, hybrid and oligomannose types of N-glycans [42,43], the current study indicated the potential of these different types of N-glycans in modulating electrical signalling in neurons.

## Conclusion

A recent study reports that N-glycans of Kv3.1b regulate cell surface expression since the cell surface expression is reduced 10-fold for the unglycosylated Kv3.1b protein compared to its glycosylated counterpart [44]. However, current density measurements of Kv3.1b expressing Sf9 insect [15], NB [17], NB_1, and NB_1 (-Mgat2) cell lines did not detect significant differences in cell surface expression between the glycosylated and unglycosylated Kv3.1b channels. The discrepancy between the studies could be due to the use of generating Kv3.1b-expressing cells by transient transfection [30] versus stable transfection (Figure 2) since transient transfections generate immature Kv3.1b glycoprotein. Nonetheless, the level of outward ionic currents, along with the TIRF microscopy results, of Kv3.1b-expressing cells supported that significant levels of Kv3.1b protein without N-glycans are trafficked to the cell surface in NB cells and primary adult mammalian neurons.

N-glycans are critical for the development and maintenance of multicellular organisms [7,45], and irregular N-glycosylation processing is commonly related to neurological problems as highlighted by CDG [4,5]. Our current study showed that processing of N-glycans, along with N-glycosylation occupancy, are critical determinants in modulating the sub-plasma membrane distribution of Kv3.1b and the opening and closing rates of the Kv3 channels containing Kv3.1b. Taken together, we suggest that N-glycosylation processing of Kv3.1b in Kv3 channels is a contributor of neural firing activities, and irregularities in N-glycosylation processing of Kv3.1b may contribute to neurological difficulties.

## Abbreviations:

Kv: Voltage Gated Potassium Channel
NB: Neuroblastoma
TIRF: Total Internal Reflection Fluorescence
DIC: Differential Interference Contrast
GlcNAcT: N-Acetyl Glucosyl Aminyl Transferase
CDG: Congenital Disorders of Glycosylation
WT: Wild Type
L-PHA: *Phaseolus vulgaris* Leucoagglutinin
GNL: *Galanthus nivalis* Lectin
E-PHA: *Phaseolus vulgaris* Erythroagglutinin
ConA: Concanavalin A
